# Geometrically complex 3D-printed phantoms for diffuse optical imaging

**DOI:** 10.1364/BOE.8.001754

**Published:** 2017-02-23

**Authors:** Laura A. Dempsey, Melissa Persad, Samuel Powell, Danial Chitnis, Jeremy C. Hebden

**Affiliations:** Medical Physics and Biomedical Engineering Department, University College London, WC1E 6BT, London, UK

**Keywords:** (170.6960) Tomography, (170.0110) Imaging systems, (170.6920) Time-resolved imaging, (170.3880) Medical and biological imaging

## Abstract

Tissue-equivalent phantoms that mimic the optical properties of human and animal tissues are commonly used in diffuse optical imaging research to characterize instrumentation or evaluate an image reconstruction method. Although many recipes have been produced for generating solid phantoms with specified absorption and transport scattering coefficients at visible and near-infrared wavelengths, the construction methods are generally time-consuming and are unable to create complex geometries. We present a method of generating phantoms using a standard 3D printer. A simple recipe was devised which enables printed phantoms to be produced with precisely known optical properties. To illustrate the capability of the method, we describe the creation of an anatomically accurate, tissue-equivalent premature infant head optical phantom with a hollow brain space based on MRI atlas data. A diffuse optical image of the phantom is acquired when a high contrast target is inserted into the hollow space filled with an aqueous scattering solution.

## 1. Introduction

Objects which mimic the optical properties of human tissues, known as phantoms, are commonly used to evaluate the performance of devices built for diagnostic applications of near-infrared (NIR) spectroscopy and imaging [1]. Light propagation in biological tissues at NIR wavelengths is dominated by elastic scattering, although the diagnostic information normally stems from the strong variation in absorption, particularly by the oxygenated and deoxygenated forms of hemoglobin in blood [2]. For most applications, it is sufficient for phantoms to mimic the transport (or “reduced”) scattering coefficient (denoted by μ_s_′, with units of mm^−1^) and the (highly wavelength-dependent) absorption coefficient (μ_a_, also with units of mm^−1^). Over many years, a large variety of recipes have been devised for constructing phantoms in both liquid and solid form, each often involving mixing a scattering agent (e.g. powders and microspheres) and absorbing pigment (e.g. molecular dyes and inks) within a matrix such as water, gels, epoxy resins, or silicone rubbers [1, 3].

The imaging technique known as diffuse optical tomography (DOT) involves coupling multiple sources and detectors to the skin in order to generate a three-dimensional (3D) image representing the optical properties of the underlying tissues. The vast majority of DOT research has focused on imaging changes in blood volume and oxygenation within the brain, and during the past five years DOT has flourished as a tool for the study of brain function and neuropathology, acute brain injury, and neurodevelopment [4, 5]. Assessing improvements in sensitivity and spatial resolution of DOT as the methodology and associated instrumentation have advanced calls for increasingly sophisticated phantoms. Constructing tissue-like objects with geometries as complex as the brain is very challenging using conventional mold-making and casting techniques, and consequently there has been a growing interest in utilizing 3D printing (“rapid prototyping”) technology. For example, Sheng *et al* [6] have described the manufacture of multi-layer skin-simulating optical phantoms using a bespoke 3D printer. Meanwhile Bentz *et al* [7] report the use of 3D printing to generate a phantom in the form of a whole rat with heterogeneous optical properties. However to our knowledge no one has yet proposed a method of 3D printing large geometries with customizable and precisely known optical properties.

At UCL, DOT is being developed primarily as a tool for imaging the brain of infants [8, 9]. Sophisticated 32-channel time-resolved DOT systems have been constructed at UCL which acquire measurements of the flight times of NIR photons across the infant head in order to produce whole-brain images which exhibit pathology such as intraventricular hemorrhage and ischemic stroke, or dynamic behavior such as seizures, adjustments to ventilator settings, and evoked responses to stimuli [10, 11]. Evaluating the imaging performance of such systems (such as sensitivity, spatial and temporal resolution, single-to-noise ratio, and susceptibility to artefacts, etc.) requires phantoms with precisely known properties and geometries, yet must also replicate the infant head as far as possible. To address this need, a means of generating an anatomically accurate premature infant head with tissue-like optical properties has been developed using a low-cost, commercial 3D printer. The phantom design and production process is presented in the following sections, and an experiment performed to image the phantom is briefly described.

## 2. Methods

### 2.1 Creating a recipe for 3D printed phantoms

The 3D printer selected to produce printed solids with tissue-like optical properties is a Form 1+ stereolithography (SLA) printer (Formlabs, USA) which uses a clear UV-cured methacrylate photopolymer resin (formulation FLGPCL02). To confirm that the resin could provide a thick, homogenous, optically transparent matrix, a single block of dimensions 15 mm × 15 mm × 40 mm was printed. This was then sliced into thin segments using a miniature band saw, wet polished, and inspected under a microscope to confirm the absence of bubbles or significant defects.

The next step to developing a phantom recipe was to determine the characteristic absorption and refractive index of the clear solid resin. To measure the absorption, liquid resin was inserted into four transparent cuvettes of thickness 20 mm, 25 mm, 30 mm, and 35 mm, custom-made from laser-cut acrylic sheet. The resin was UV cured and each cuvette was inserted into a NIR spectrometer (PerkinElmer, USA) to measure the transmittance between 650 nm and 950 nm. The slope of the linear regression for the natural log of the transmittance plotted against thickness yields the μ_a_ of the resin as a function of wavelength. The refractive index *n* of the resin at a wavelength of 630 nm was estimated to be 1.46 ± 0.03 by measuring the deviation of a laser beam transmitted across a parallel-sided block with a thickness of 15 mm, averaged over eight angles of incidence in the range 10° - 80°.

To provide tissue-like scattering, we selected a titanium dioxide (TiO_2_) based polyester pigment (Superwhite, Tiranti Ltd., UK) which we have used previously for phantoms based on epoxy resin [12]. Other scattering pigments could also be used, such as polymer microspheres or quartz glass microspheres [1]. Since the refractive indices of the clear FLGPCL02 resin and our previous epoxy resin were similar, it was initially assumed that similar concentrations of pigment should yield approximately the same transport scattering coefficients μ_s_′. A series of five solid blocks (15 mm × 15 mm × 40 mm) were printed, with different concentrations of TiO_2_ pigment added to the resin, to achieve estimated values of μ_s_′ in the range 0.8 mm^−1^ to 3.0 mm^−1^. In each case, a carefully weighed amount of pigment was added to a known mass of resin, and then thoroughly mixed by hand. Our trials with clear samples of resin indicated that vacuum de-gassing to remove air bubbles was not required, despite stirring. This is unlike the use of most epoxy resins or silicone rubbers which have a higher viscosity. Furthermore, the period between mixing and hardening can be hours or days, since unlike epoxy resins there is no requirement to stir in a chemical catalyst immediately before the hardening occurs.

The actual values of μ_s_′ were then obtained by acquiring measurements of the time-resolved transmittance across each block using the UCL time-resolved wavelength-tunable DOT system known as MONSTIR II [10]. Each block was encased in an opaque box, which supports optical fibers in contact with opposite surfaces of the block, aligned across a 15 mm thickness. The box was also 3D printed, using another 3D printer which uses a NIR-opaque black resin (VeroBlack material on the Objet Scholar, Stratasys, MN, USA). Histograms of photon flight-times (known as temporal point spread functions, or TPSFs) transmitted across each block were acquired while illuminating using picosecond pulses of light at discrete wavelengths from 650 nm to 850 nm at 50 nm intervals. Finally, values of μ_s_′ were derived by fitting a Green’s function solution to the time-dependent diffusion equation (convolved with a measurement of the system impulse response function) to the measured TPSFs [13], using the pre-calculated values of μ_a_ and a refractive index *n* = 1.46 (assumed to a first-order to be wavelength independent). The μ_s_′ values were plotted against pigment concentration, and a linear regression performed. The slope of the regression was then plotted against wavelength and another linear regression calculated to yield a simple equation that could be used to tailor μ_s_′ at any wavelength in the NIR.

Adjusting the absorption of the phantom material can be achieved by adding a compatible, non-scattering molecular dye. We confirmed the compatibility of the NIR dye used for previous epoxy resin phantoms (Projet 900NP, ICI, UK) by mixing controlled amounts into the 3D printer resin. The dye was originally obtained in a powdered form, and mixed within a small amount of uncured polyester resin to produce a liquid concentrate with a pre-calibrated absorption spectrum. The intrinsic absorption spectrum of the dye has been published previously by Firbank *et al*. [12]. Four 1 cm × 1 cm cuvettes were filled with resin and absorber in different concentrations, which were then UV cured. Transmission spectra were recorded using the NIR spectrometer, and a linear regression was used to estimate μ_a_ as a function of concentration over a range of NIR wavelengths.

### 2.2 Printing an anatomically accurate phantom

**Fig. 1:**
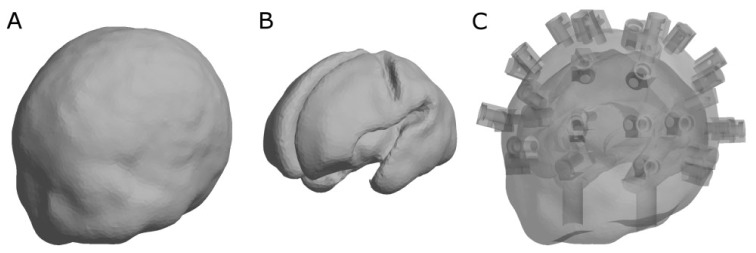
Progress of the CAD model used to make the 3D printed, anatomically-accurate phantom. An atlas derived from MRI scans for a 29 week old infant was used to create the phantom [14]. A) scalp surface, B) combined white matter and grey matter surface, and C) transparent render of final CAD model with hollow brain space, temporal ducts and fiber holders at specific 10-5 scalp locations [15–17].

To illustrate the considerable potential of 3D printing for phantom construction, we chose to develop a phantom in the form of an infant head and brain geometry, selected from a neonatal head atlas created from MRI scans of infants with gestational ages ranging between 29 and 44 weeks [14]. The smallest available head size (29 weeks) was chosen due to the size constraints of the Form 1+ printing bed. The scalp surface mesh and combined white matter/grey matter surface mesh were converted to Standard Tessellation Language (STL) format and then made into STandard for Exchange of Product (STEP) solids using FreeCAD (http://www.freecadweb.org/). The brain solid part was Boolean subtracted from the scalp solid part, and temporal ducts were cut away from the resulting combined solid (these allow the user to fill the phantom with liquid and insert targets). To aid attachment of optical fiber bundles for imaging the phantom, 32 fiber holders were added directly to the printed volume at locations corresponding to the 10-5 placement convention which we have used for previous DOT infant studies [15–17]. Each fiber holder included a 2 mm hole to accommodate a grub screw. The progression of the phantom design and final CAD model is illustrated in Fig. 1

. The final CAD model was divided into four pieces for printing so that the support structures within the internal cavities of the phantom could be manually removed later.

The results of the recipe development (see Results below) were applied to calculate the concentrations of TiO_2_ pigment and NIR dye required to generate a printed solid with properties of μ_s_′ = 1.0 mm^−1^ and μ_a_ = 0.04 mm^−1^ at a wavelength of 800 nm, which are typical of the newborn infant brain [18–20]. The appropriate amounts of pigment and dye were added to the clear resin, manually mixed, and poured into the build tray of the Form 1+ printer. The phantom was printed in four parts, each with the resin type set to “grey” in the Formlabs PreForm software. After completion of the print, the support structures on the part were removed with small wire cutters, washed in isopropyl alcohol and lightly sanded with abrasive paper. The four parts were then glued together using with a small amount of polyester resin (Tiranti Ltd., UK) to which were added small amounts of catalyst hardener, TiO_2_ pigment, NIR dye, and Thixotropic Paste (Tiranti Ltd., UK). The hole on each fiber holder was manually tapped and a M2.5 black nylon screw inserted.

### 2.3 Imaging the phantom

To verify that the 3D printed infant head is effective as a phantom for DOT imaging, a simple imaging experiment was performed using the UCL time-resolved system, MONSTIR II [10]. The phantom was supported upside down, and the brain cavity was filled via the temporal ducts [Fig. 1] with a mixture of intralipid and an aqueous solution of NIR dye with optical properties μ_a_ = 0.01 mm^−1^ and μ_s_′ = 1.0 mm^−1^ at 800 nm. The 32 fiber bundles of the imaging system (each incorporating a detector bundle and a co-axial source fiber) were attached to the phantom’s fiber holders. Time-resolved data were then recorded before and after an optically black cylindrical target (diameter 8 mm, height 12.5 mm) was inserted into the right parietal region via the temporal duct on the right side. The data consist of TPSFs at a wavelength of 800 nm for each source-detector pair (i.e. 1024 distinct histograms), with and without the target inserted. Each source was illuminated for 2 seconds at a time for 10 cycles total.

Prior to image reconstruction, the data were filtered to remove any contamination by ambient light, and 21% of the TPSFs were removed completely due to either exceptionally low photon counts, artefacts due to multiple reflections, or other causes of systematic error [21, 22]. Specifically, log of the total photon count (log intensity) difference data with a standard deviation greater than 0.04 and mean flight-time (meantime) difference data with a standard deviation greater than 8 ps were removed. Finally, values of meantime and log intensity were calculated for each remaining TPSF. The TOAST++ image reconstruction package [23] uses a mesh of the phantom to describe the geometry of the forward problem; in this case it was identical to that used to generate the CAD file. Jacobians (sensitivity matrices) for log intensity and meantime datatypes were calculated by solving the diffusion approximation by the finite element method. The sensitivity matrices were calculated assuming homogenous background optical properties of *n* = 1.46, μ_a_ = 0.02 mm^−1^, and μ_s_′ = 1.0 mm^−1^, the average of the experimental values for the solid resin and the intralipid solution. To improve computational performance, the Jacobians were mapped from the volumetric mesh space to a 40 × 40 × 40 grid basis. Both the data and their respective Jacobian were normalized by the variance of the data and weighted by the Euclidean distance between channels (expressed as a fraction of the maximum distance). The Jacobian was augmented with zeroth-order Tikhonov regularization, prior to the calculation of its pseudoinverse. The regularization parameter was set equal to 0.005 (i.e. 0.5% of the maximum of the singular value decomposition of the Jacobian). An image representing the change in μ_a_ was created by multiplying this inverted and regularized Jacobian by the differences in mean time and log intensity (i.e. with and without the target), and then mapping back from the basis to the volumetric mesh for display.

## 3. Results

### 3.1 3D printed phantom recipe

**Fig. 2:**
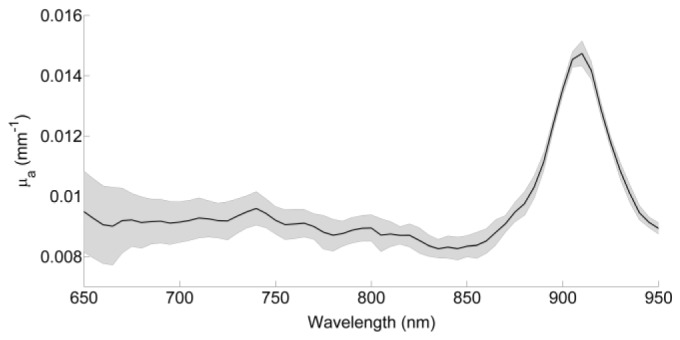
Absorption coefficient as a function of wavelength ( ± standard deviation of three trials) for the Formlabs clear FLGPCL02 resin.

The relationship between μ_a_ and wavelength for the Formlabs clear FLGPCL02 resin is shown in Fig. 2

, derived from a linear fit to the attenuation measured across four different thicknesses of resin. Within the 650 nm – 850 nm region the spectrum is relatively flat (R^2^ ≥ 0.95 for all wavelengths). These values are lower than the typical absorption coefficients of most tissues, and thus tissue equivalence can be achieved by adding a suitable absorbing dye to the resin. Beyond 850 nm μ_a_ markedly increases, but this is outside the wavelength range typically used by commercial NIRS systems.

**Fig. 3:**
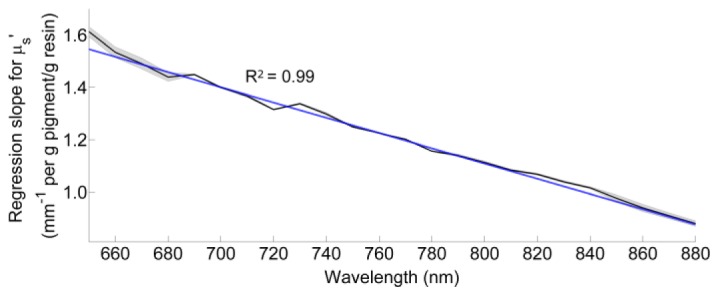
Slope of the linear regression between transport scattering coefficient and concentration for wavelengths in the range of 650 – 880 nm. Experimental standard deviation from the TPSF acquisition is included.

When TiO_2_ pigment was added to the resin, the value of μ_s_′ derived from the Green’s function fit to the TPSF was observed to increase linearly with concentration at each wavelength. Figure 3

shows the slope of μ_s_′ vs. concentration for each wavelength, obtained from fits of the Green’s function to the measured TPSFs recorded across the resin samples with five different concentrations of TiO_2_ pigment (ranging from 0.001 mm^−1^ to 0.004 mm^−1^). Linear regression yields the relationship:

$$\left[TiO_{2}\right]=\frac{\mu_{s}{'}_{\lambda }}{3.4\times 10^{3}-2.9\lambda }$$ (1)

with R^2^ = 0.99, where [*TiO_2_*] is the mass (g) of scattering pigment (i.e. Superwhite) per g clear resin, *λ* is the wavelength (nm), and *μ_s_′_λ_* is the transport scattering coefficient at the same wavelength (mm^−1^). For example, to obtain a μ_s_′ = 1.0 mm^−1^ at 800 nm, 9.3 × 10^−4^ g scattering pigment per g resin are required.

The Projet dye spectrum has been published previously [12]. We observed no indication of the spectrum of the dye being different whether it was assessed independently or incorporated into the clear resin. The μ_a_ of the mixture used for 3D printing will be equal to the μ_a_ of the clear resin at a particular wavelength, plus the μ_a_ of the user’s chosen NIR dye at that same wavelength. Analogous to calculating the [*TiO_2_*] needed for a certain μ_s_′, the absorption coefficient of the mixture can be characterized as a function of NIR dye concentration. For our chosen NIR dye, at 800 nm this leads to a relationship of

$$[dye]=\frac{\mu_{a800nm}-0.0085}{28.1}$$ (2)

where [*dye*] is the concentration of our Projet dye solution (g dye/g resin) and *μ_a_*_800_*_nm_* is the desired absorption coefficient of the final phantom at 800 nm (mm^−1^). We observed a very linear relationship (R^2^ = 1.0) for this regression, demonstrating the stability of the dye in the resin. The robustness of these data is verified by the y-intercept (rearranged to be in the numerator of Eq. (2)), which is equal to the intrinsic absorption coefficient of the base clear resin at 800 nm (0.0089 mm^−1^ ± 0.0002).

#### 3.2 3D printing

The four components of the phantom were printed consecutively on the same Formlabs printer, but the success rate was about 1 in every 10 attempts. This was mostly due to the peeling mechanism of the Form 1+ being insufficient, leading to a build-up of material curing to the base of the resin tray as opposed to the correct layer on the build platform. The problem was independent of our customized mixture, as we also observed this when printing with pure FLGPCL02 resin.

**Fig. 4:**
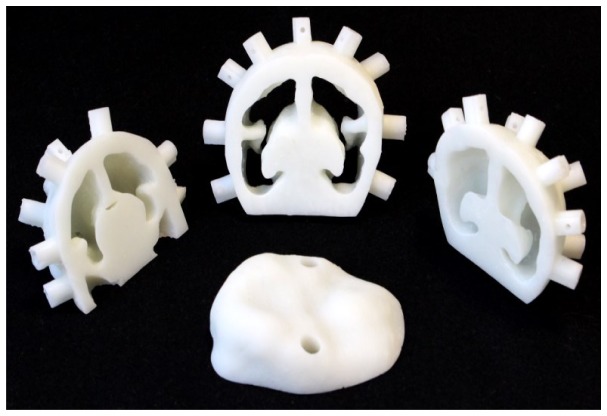
The premature infant head phantom was printed in four pieces so that the hollow brain space could be cleaned of support structures. Optical properties are μ_a_ = 0.04 mm^−1^ and μ_s_′ = 1.0 mm^−1^.

**Fig. 5:**
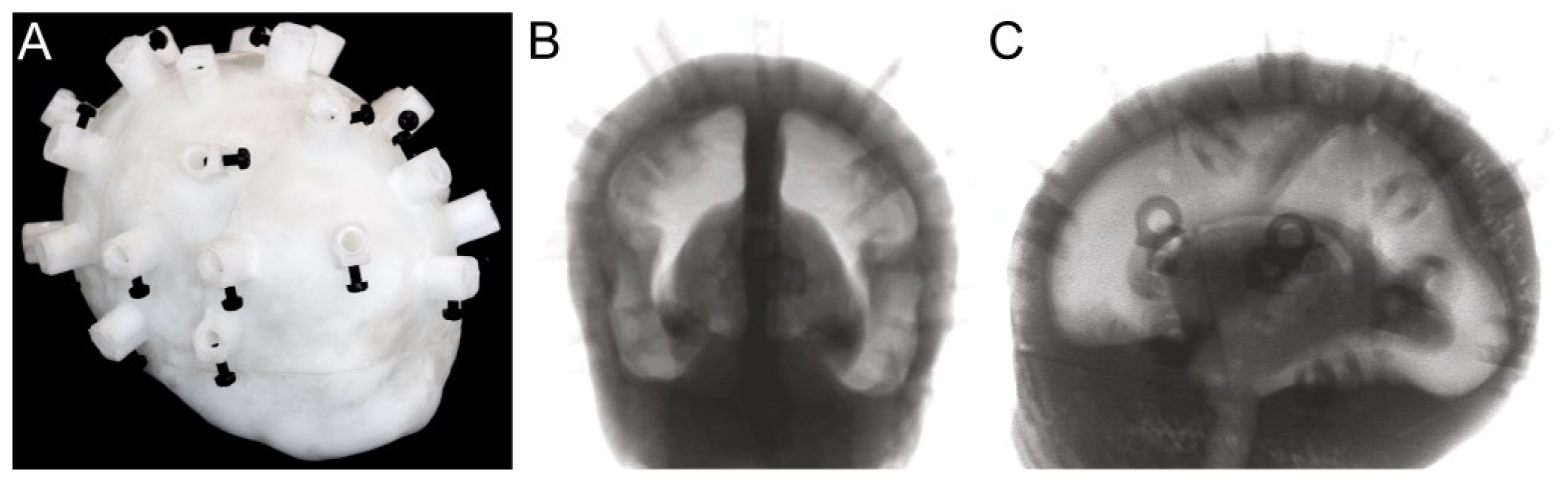
A) Final assembled phantom with grubscrews to hold the fiber optic cables, B) coronal and C) sagittal x-ray of the final assembled phantom, showing the brain cavity and temporal ducts. Subtle lines can be seen in the sagittal view, showing where the four phantom pieces were joined.

The four components of the phantom, after removing support structures and cleaning, are shown in Fig. 4

, and the final assembled phantom along with coronal and sagittal projection x-ray images revealing the hollow brain cavity are shown in Fig. 5

. Each piece of the phantom has optical properties of μ_s_′ = 1.0 mm^−1^ and μ_a_ = 0.04 mm^−1^ at a wavelength of 800 nm.

### 3.3 Reconstructed images

Transcranial TPSFs were acquired across the entire phantom. After objectively removing poor quality TPSFs, 79% of the data were used to perform the image reconstruction.

**Fig. 6:**
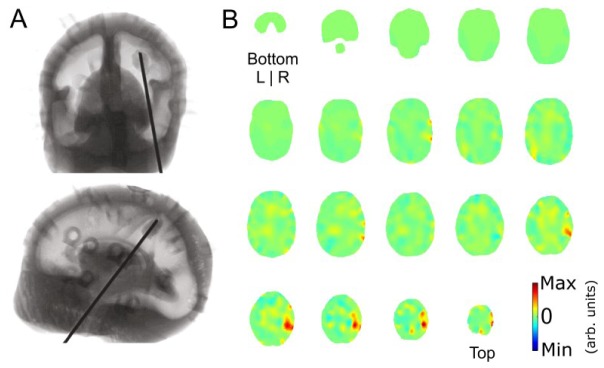
A) Coronal (top) and sagittal (bottom) x-ray images of the phantom with the target inserted. The target can be faintly seen on the end of the metal rod in the right parietal area, posterior of the central sulcus. B) Axial view of the 800 nm log intensity and meantime image starting at the bottom of the head (top left) and sliced in 4.6 mm sections to the top of the head (bottom right). The target can be seen in the upper right hemisphere (arb. units). Residual artefacts in the right hemisphere are likely due to the metal wire attached to the target.

An x-ray of the phantom with the target inserted is pictured in Fig. 6(a)

. Figure 6(b) shows a series of axial slices of the reconstructed log intensity and meantime image representing the absorption coefficient of the phantom at 800 nm. The target is visible in the upper right hemisphere in the correct location. Additional artefacts near the target in the right hemisphere are likely due to the target’s metal wire.

## 4. Discussion

A means of generating geometrically-complex tissue-equivalent phantoms using a low-cost commercial SLA printer has been demonstrated. The infant head phantom described above would have been extraordinarily challenging to fabricate using conventional mold-making and casting techniques. The recipe is simple, relatively inexpensive, and does not require additional sophisticated equipment. The addition of polyester-based scattering pigment and NIR absorbing dye to the clear base resin did not inhibit the cure, and enabled the values of μ_s_′ and μ_a_ to be adjusted in linear proportion to their concentrations. Apart from the time spent mixing these additives to the resin, the only significant tasks are designing the CAD file and a few minutes removing support structures from the printed solid.

The relatively high NIR transmission of the Formlabs transparent resin, and the lack of strong absorption features in the spectrum, makes it a convenient matrix for DOT phantoms. Although the resin was observed to yellow slightly when cured, this is accommodated within the recipe which is based on the absorption spectrum of the cured resin. The resin was readily miscible with the pigment and dye solution, and there was no evidence of bleaching of the dye.

We successfully used our 3D printed phantom to conduct an imaging experiment using both log intensity and meantime data. Despite the relatively small target size coupled with the sparse layout of sources and detectors over the head, we were still able to localize the target position accurately with a spatial resolution on the order of 1 cm. The ability to create anatomically correct and tissue-equivalent phantoms will help lead to increasingly sophisticated methods of evaluating DOT hardware and image processing techniques. Our 3D printed phantom recipe could also be used for other optical imaging modalities where scattering and absorption properties are important, such as optical coherence tomography.

The printing success rate of about 10% was disappointing and frustrating, although we anticipate that new generations of printers will prove to be much more reliable. There are several limitations of the process as described here. The Form 1+ printer has a relatively small printing bed, and is only capable of printing structures up to 125 mm × 125 mm × 165 mm (although the new Form 2 model has a printing area of 145 mm × 145 mm × 175 mm). The long term stability of the Formlabs transparent resin (especially of its optical properties) is unknown at present, and preliminary evidence suggests that it continues to yellow with age (particularly when exposed to sunlight) and become more brittle.

As with all forms of 3D printing, the shape and geometric constraints of the CAD model are a crucial factor. There are certain geometries that cannot be printed with a SLA system, such as hollow interior spaces with narrow openings, which require a technique like selective laser sintering (SLS). This problem was circumvented here by printing the phantom in multiple parts and gluing them together after cleaning.

Although the optical properties of the phantom presented were uniform, it would be relatively straightforward to print multiple components from base resins with different concentrations of scattering pigment and absorbing dye, and then assemble the components into a highly heterogeneous structure. However, the ideal 3D printer for making phantoms would be able to print spatially varying optical properties throughout the volume in a single printing session. This would allow the user to specify the optical properties of each node of the CAD model; for example, one could make a scattering brain, clear CSF layer, and scattering skull and scalp layers as opposed to the combined approach we have taken here. Systems are already commercially available that allow 3D printing using multiple materials simultaneously, and these are likely to become cheaper, faster, and more versatile as 3D printing technology continues to evolve.
